# Annual Meetings of Hospitals

**Published:** 1921-04-30

**Authors:** 


### ANNUAL MEETINGS OF HOSPITALS.

### Metropolitan Hospital.

Major Lionel de Rothschild, M.P., presided at the annual meeting of Governors, held at the hospital on April 14. He said that the Metropolitan Hospital had passed a critical stage, and has now reached an interesting period in its history. The question was, "Can hospitals carry on on the voluntary principle?" He confessed he is an optimist in that matter, and firmly believes that they can. The hospital had suffered a great loss through the death of its Secretary, Mr. Dale. He commended the proposed new rules which were put before the meeting and passed. In future the honorary medical consulting and surgical staffs will be Governors. Another new rule is one whereby the employees at any establishment contributing not less than five guineas in any one year as an established contribution shall be entitled to nominate for election any contributor for the year following. The number of in-patients during 1920 was 1,638, as compared with 1,989 in 1919. The total income for the year, which amounted to £43,875, and exceeded the expenditure by £12,568, was increased to an exceptional degree by the receipt of an emergency grant of £11,000 from the King's Fund, £2,200 from the National Relief Fund, and £6,000 from the Special Appeal Committee and other donors for the specific purpose of reducing debt. Patients' payments totalled £7,155. The Committee expressed their appreciation of the way in which Miss M. Partick discharged the duties of Secretary pending the appointment of Mr. H. E. Rutherford.

### The Anti-Vivisection Hospital.

The financial position of this hospital had recently become so acute that a special Court of Governors was held at the hospital on Monday, April 4, with a view to obtaining the necessary powers to close down many of the wards. Lord Tenterden, however, reported that owing to strenuous efforts which had been made there is now a prospect of weathering the storm. The Mayor of Battersea has issued a personal appeal. One lady patron had sent a cheque for £1,000 on the understanding that the hospital is to be kept going, and there is now a balance at the bank, and all bills have been paid. The work involves, however, an expenditure of from £700 to £1,000 every month, and the staff has had to be reduced. After hearing the Chairman's statement, the Court unanimously agreed that the question of closing down wards or otherwise curtailing the work should be deferred until the annual Court of Governors in May.

### The London Jewish Hospital.

At the fourteenth annual meeting, on Sunday, April 10, the chair was taken by Mr. I. Berliner, the President, who said that the year's work had been successful, but that the front block of the building could not be completed unless a ready response to the appeal for funds was forthcoming. The annual report stated that amongst Jewish charities the hospital, in respect of financial status, ranked as second only to the Jewish Board of Guardians. At present out-patient work only is carried out, and the daily attendance averaged 120. Patients are seen on four mornings and five afternoons a week, and there is a special eye-clinic for school children. During 1920 the minor operations performed in the out-patient department numbered 250. The income of the year was £14,749, as against £7,049 in 1919. The surplus income over expenses was £8,674, as against £7,761 in 1919, but the funds in hand were not yet sufficient to complete the in-patient scheme and the nurses' home which are in prospect.

### Cancer Hospital.

Lord Northbrook presided at the annual meeting. Sir Chas. Ryall, the Senior Surgeon and Chairman of the Medical Committee, said that the Registrar-General's figures, showing a greater number of deaths from cancer than in the preceding year, were causing a considerable amount of anxiety. No doubt the war and the consequent anxiety had been a severe strain on the people of this country, lowering their resisting power and making them an easy prey to malignant diseases. At the same time, it was quite possible, on analysing the figures, one might derive satisfaction from the fact that doctors throughout the country were gaining a greater knowledge of cancer and how to diagnose it, and it was by knowledge alone that the malady could be fought. New in-patients numbered 810, as compared with 631 in 1919. Out-patients totalled 1,127, as against 811. The total number of operations was 616; of those, 226 were abdominal, with a mortality of only 3 per cent. During the year the pathological and research departments had been reorganised and a well-equipped dental department established. In order to expand and increase the efficiency of the staff, arrangements had been made to foster more intimate collaboration between physician, surgeon, and radiologist.

